# Supplementary figures and images for: Separable, Ctf4-mediated recruitment of DNA Polymerase α for initiation of DNA synthesis at replication origins and lagging-strand priming during replication elongation
Source: PLoS Genet. 2020 May 7;16(5):e1008755. doi: 10.1371/journal.pgen.1008755 (PMC7237047; doi:10.1371/journal.pgen.1008755)

Porcella et al., Figure S1, associated with Figure 1

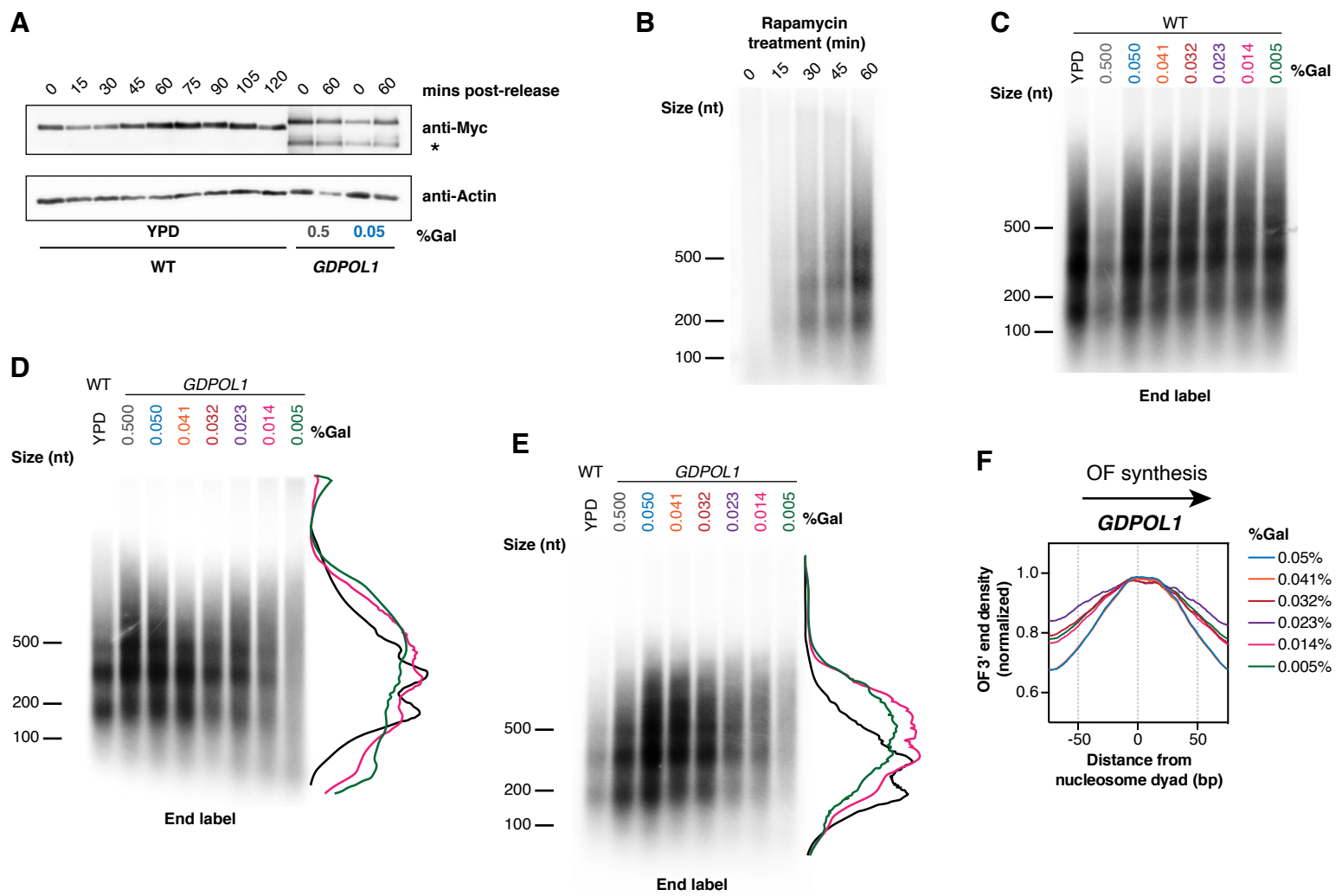

Supplement: S1 Fig — (A). Western blot against 13xMyc-tagged Pol1 from wild-type or GDPOL1 cells at the indicated sugar concentration, released from alpha-factor arrest for the indicated time. The GDPol1-specific degradation product is indicated by an asterisk. (B). Timecourse of Okazaki fragment enrichment during Cdc9 nuclear depletion by anchor away [32]. Okazaki fragments were prepared and labeled as in Fig 1C, and as previously described [13]. (C-E). Representative replicate Okazaki fragment end-labeling gels for wild-type (C) and GDPOL1 (D-E) at the indicated galactose concentrations. Traces adjacent to the plots in D&E indicate the change in size distribution of Okazaki fragments at 0.014% (pink) and 0.005% (green) galactose. (F). Distribution of Okazaki fragment 3’ ends around consensus nucleosome dyads [56] in the GDPOL1 strain shifted to media containing the indicated concentration of galactose. (PDF) [file pgen.1008755.s001.pdf]

Porcella et al., Figure S3, associated with Figure 1

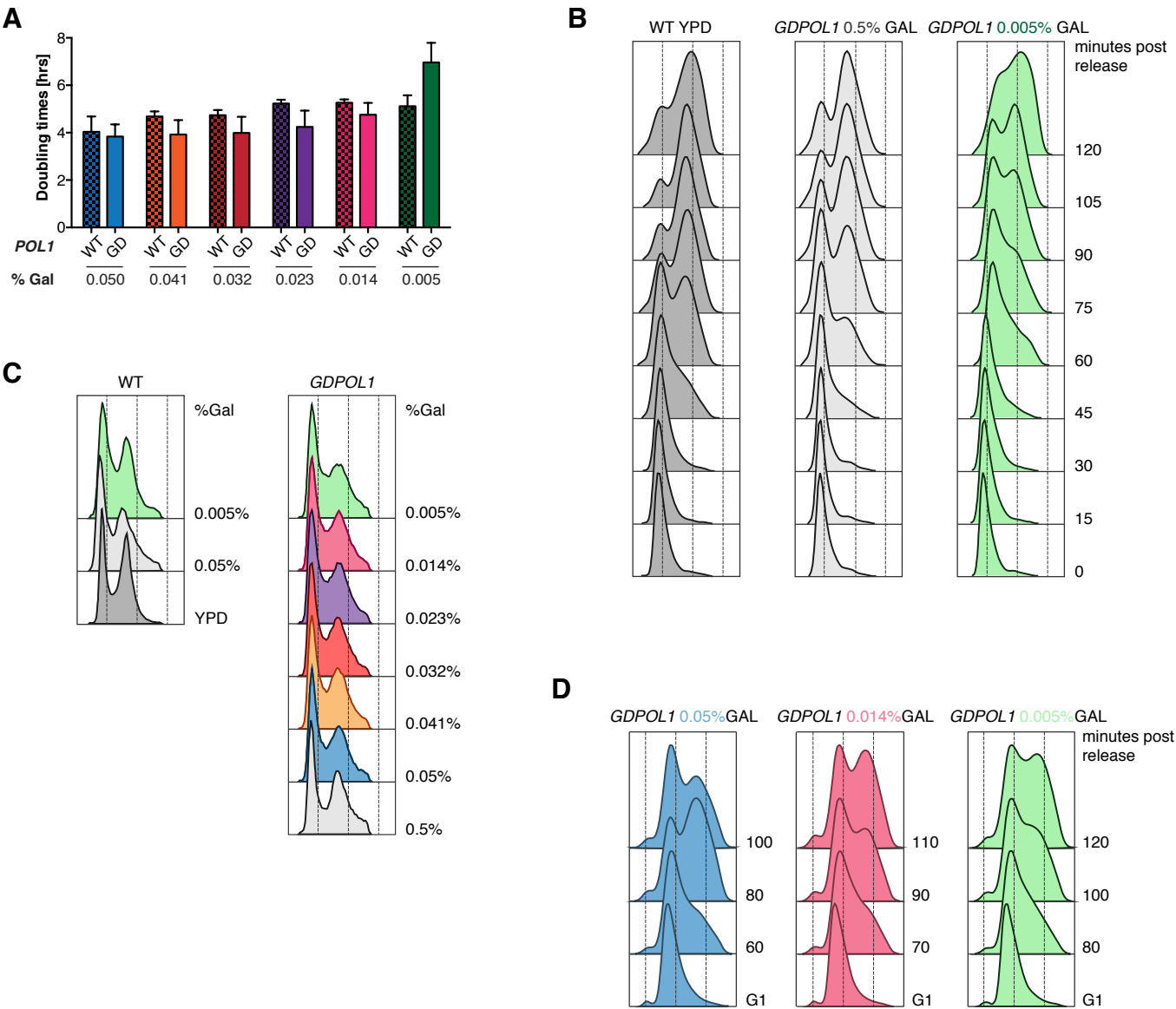

Supplement: S3 Fig — (A). Doubling times for wild-type or GDPOL1 strains in YEP + 3% raffinose, supplemented with the indicated concentration of galactose. Data are the average of at least three replicates in each case. (B). DNA content, assayed by flow cytometry, of an arrest release of wild type or GDPOL1 cells also analyzed in A. (C). DNA content, assayed by flow cytometry, of asynchronous cells post 4h sugar switch of wild type or GDPOL1 cells. (D). DNA content, assayed by flow cytometry, GDPOL1 cells, released into S-phase after 4h sugar switch in G1. The samples collected at these time points were used to generate sequencing libraries for the analysis shown in Fig 3. (PDF) [file pgen.1008755.s003.pdf]

Porcella et al., Figure S4, associated with Figure 2

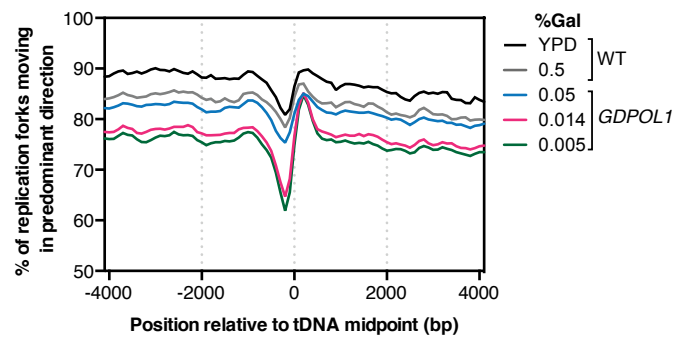

Supplement: S4 Fig — Increased replication-fork stalling or arrest at these sites would manifest as a decrease at or after the midpoint of the gene [38]. (PDF) [file pgen.1008755.s004.pdf]

2D gels: Replicate 2

0.05% Gal

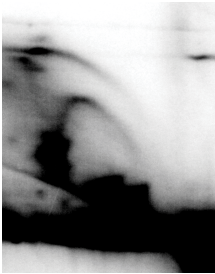

0.005% Gal

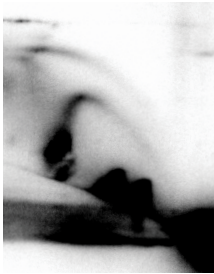

Supplement: S5 Fig — Representative replicate 2D gels of asynchronous cultures shifted to slightly (0.05% Gal) or severely depleted (0.005% Gal) Pol1 conditions. Southern blots of rDNA locus digested with StuI and probed for RDN18. These were also used in calculations done for Fig 3E. (PDF) [file pgen.1008755.s005.pdf]

Porcella et al., Figure S6, associated with Figure 4

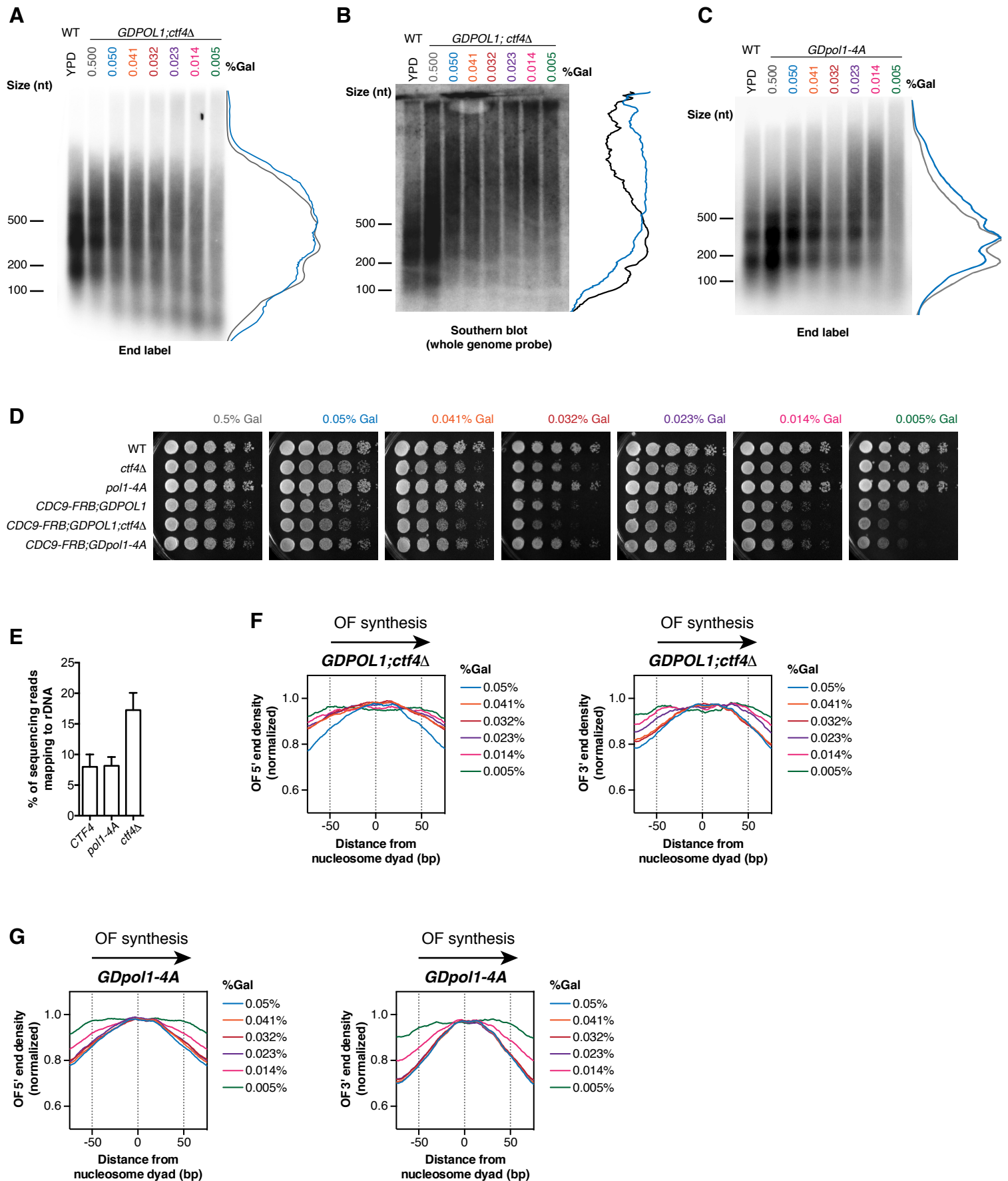

Supplement: S6 Fig — (A-C). Representative replicate end-labeling gel (A, C) or Southern blot (B), on Okazaki fragments from a GDPOL1;ctf4Δ (A, B) or GDpol1-4A (C) strain shifted to media shifted to low galactose concentrations. Traces of YPD (black) and 0.05% galactose (blue) lanes on the right. A control lane for wild-type cells grown in YPD is included on each gel. (D). Serial dilution spot tests to assay the growth of GDPOL1 strains with or without FRB tagging of CDC9 and/or ctf4Δ or pol1-4A mutations. (E). The rDNA repeat is expanded in ctf4Δ; GDPOL1 cells. The proportion of sequencing reads mapping to the rDNA is indicated. Data represent the mean ± SD of all sequencing datasets used for analysis in Figs 2&3. (F-G). Distribution of Okazaki fragment 5’ (left panel) and 3’ ends (right panel) around consensus nucleosome dyads [56] in the GDPOL1;ctf4Δ (F) or GDpol1-4A strain (G) shifted to media containing various galactose concentrations. (PDF) [file pgen.1008755.s006.pdf]

Porcella et al., Figure S9, associated with Figure 5

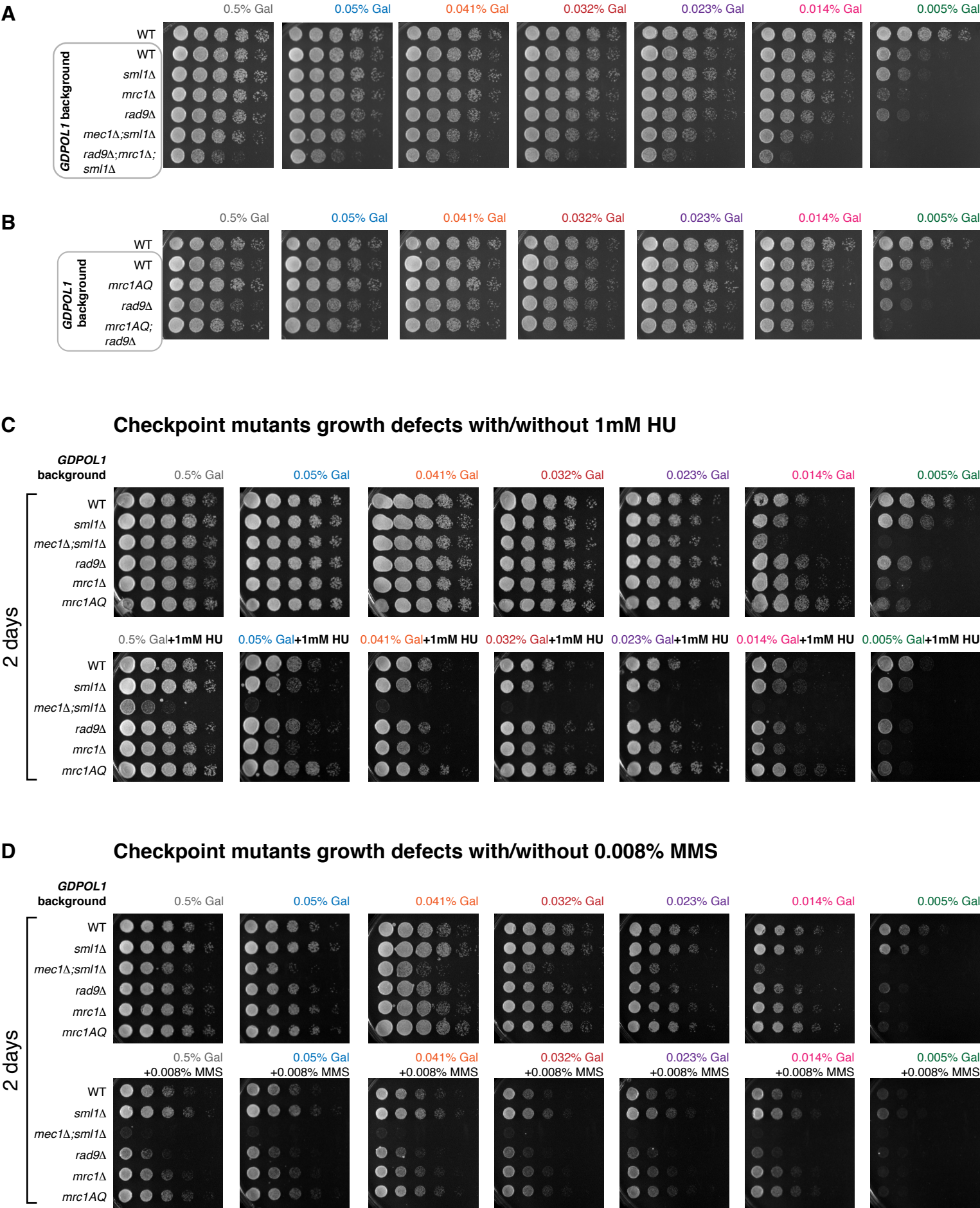

Supplement: S9 Fig — (A, B). Serial dilution spot tests to assay the growth of GDPOL1 strains carrying additional mutations (mec1Δ;sml1Δ, rad9Δ, mrc1Δ, mrc1AQ) at the indicated galactose concentrations. A selection of these concentrations is shown in Fig 4A and 4B (C,D). Serial dilution spot tests of the indicated strains with or without 1 mM hydroxyurea (C) or with or without 0.008% methyl methanesulfonate (D). Note that the full ranges of galactose concentrations were independently plated as loading/growth controls for both C and D. (PDF) [file pgen.1008755.s009.pdf]
