# Supplementary material for: Separable, Ctf4-mediated recruitment of DNA Polymerase α for initiation of DNA synthesis at replication origins and lagging-strand priming during replication elongation
Source: PLoS Genet. 2020 May 7;16(5):e1008755. doi: 10.1371/journal.pgen.1008755 (PMC7237047; doi:10.1371/journal.pgen.1008755)

Porcella et al., Figure S2, associated with Figure 2

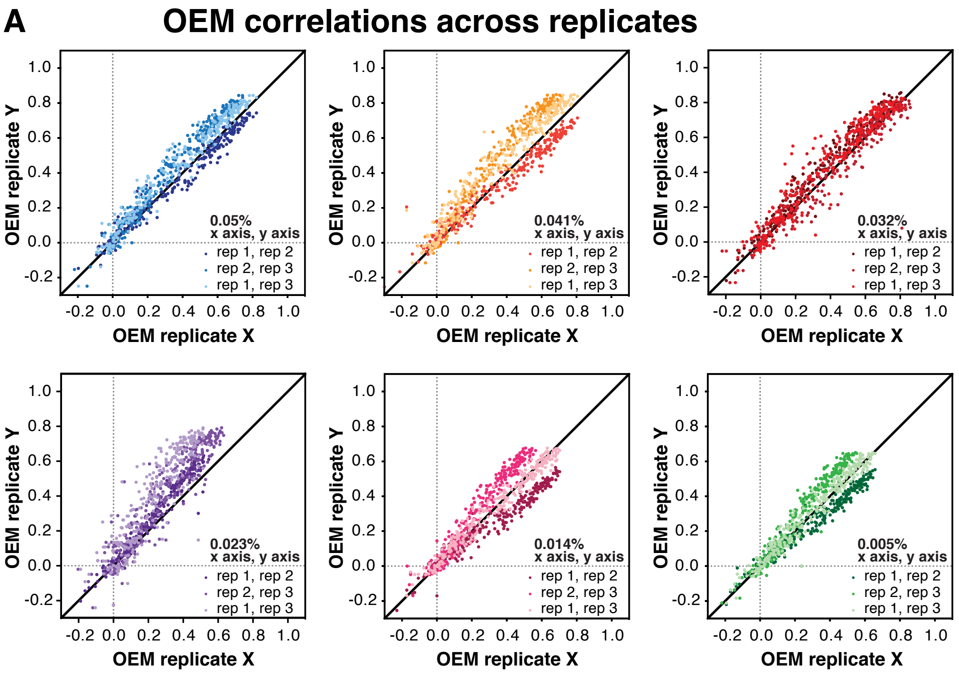

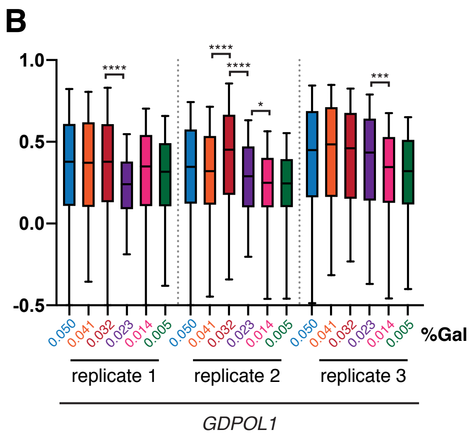

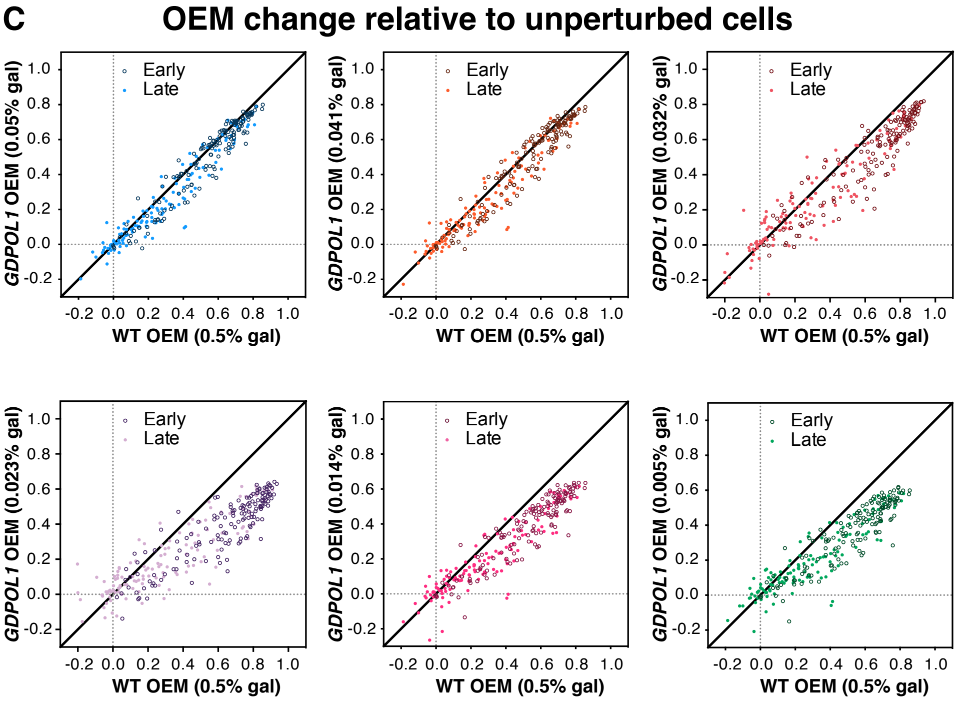

Supplement: S2 Fig — (A). Origin efficiency replicate comparisons for data from the GDPOL1 strain shown in Fig 2B each of three replicates are plotted against each other and indicated by color. (B). Comparison of origin efficiency data from each replicate from GDPOL1 cells shifted to the indicated concentration of galactose. Significance was calculated by unpaired t-test; **** p<0.0001, * p<0.05. (C). Scatter plots comparing origin firing efficiency in GDPOL1 cells at various galactose concentrations, to wild-type cells in 0.5% galactose. (PDF) [file pgen.1008755.s002.pdf]
