# Supplementary material for: Separable, Ctf4-mediated recruitment of DNA Polymerase α for initiation of DNA synthesis at replication origins and lagging-strand priming during replication elongation
Source: PLoS Genet. 2020 May 7;16(5):e1008755. doi: 10.1371/journal.pgen.1008755 (PMC7237047; doi:10.1371/journal.pgen.1008755)

Porcella et al., Figure S7, associated with Figure 4

A **OEM correlations across replicates (*GDPOL1*, *ctf4*Δ)**

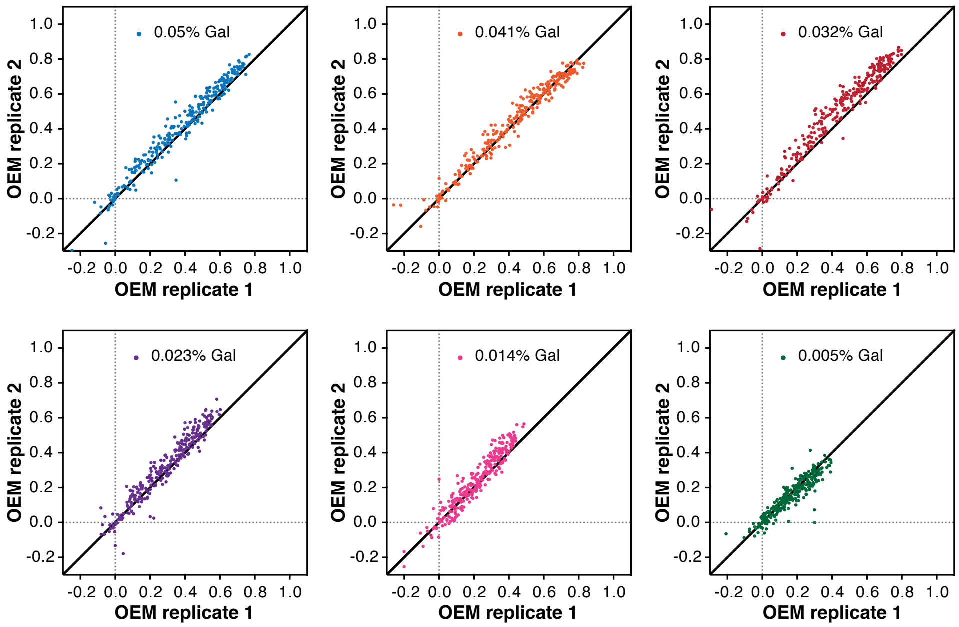

B **OEM correlations across replicates (*GDpol1-4A*)**

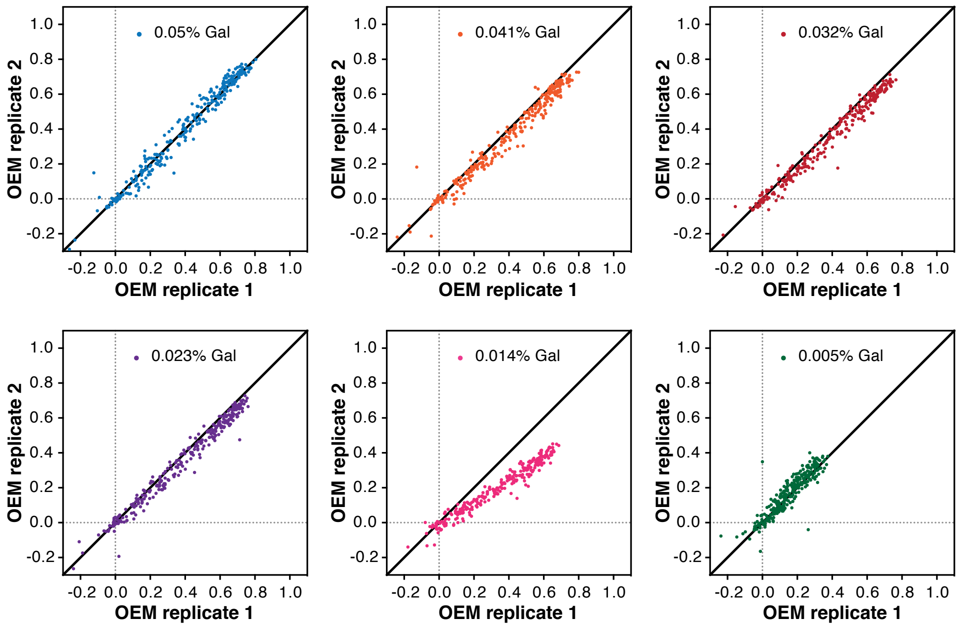

Supplement: S7 Fig — (A, B). Origin efficiency replicate comparisons for data from the ctf4Δ;GDPOL1 strain shown in Fig 3D (A) and the GDpol1-4A strain show in Fig 3F (B). (PDF) [file pgen.1008755.s007.pdf]
