# Supplementary material for: Separable, Ctf4-mediated recruitment of DNA Polymerase α for initiation of DNA synthesis at replication origins and lagging-strand priming during replication elongation
Source: PLoS Genet. 2020 May 7;16(5):e1008755. doi: 10.1371/journal.pgen.1008755 (PMC7237047; doi:10.1371/journal.pgen.1008755)

Porcella et al., Figure S8, associated with Figure 4

A. *GDPOL1-4A*

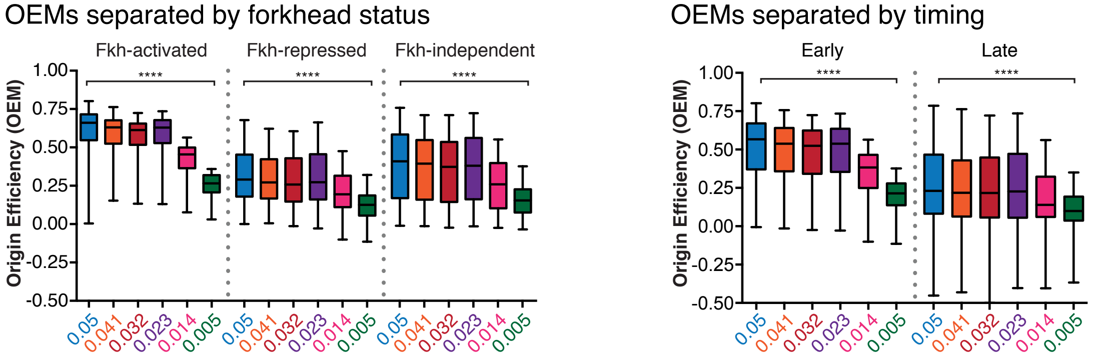

B. *GDPOL1; ctf4Δ*

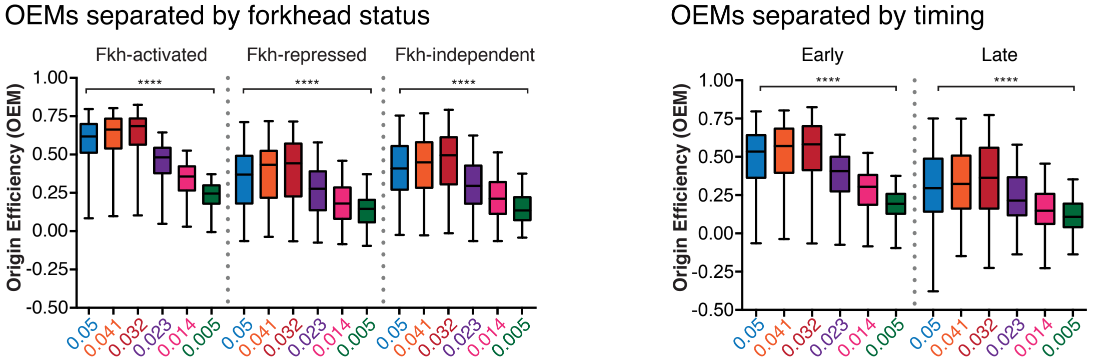

C. WT

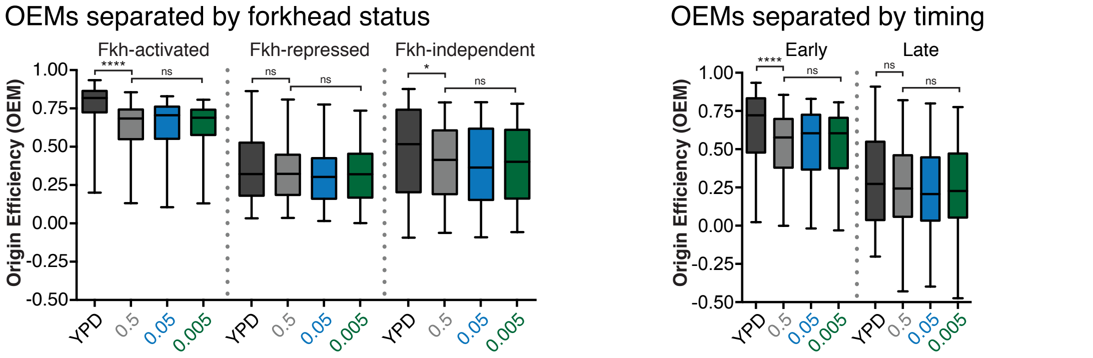

Supplement: S8 Fig — (A, B, C). Firing efficiency for origins separated by Fkh status or replication timing for the data sets in Fig 4. Significance was calculated by unpaired t-test; **** p<0.0001, * p<0.05. (PDF) [file pgen.1008755.s008.pdf]
